# Impact of Temporal pH Fluctuations on the Coexistence of Nasal Bacteria in an *in silico* Community

**DOI:** 10.3389/fmicb.2021.613109

**Published:** 2021-02-10

**Authors:** Sandra Dedrick, M. Javad Akbari, Samantha K. Dyckman, Nannan Zhao, Yang-Yu Liu, Babak Momeni

**Affiliations:** ^1^Department of Biology, Boston College, Chestnut Hill, MA, United States; ^2^Channing Division of Network Medicine, Brigham and Women’s Hospital, Boston, MA, United States

**Keywords:** microbial communities, variable environment, nasal microbiota, mathematical model, species interaction network, community ecology, coexistence

## Abstract

To manipulate nasal microbiota for respiratory health, we need to better understand how this microbial community is assembled and maintained. Previous work has demonstrated that the pH in the nasal passage experiences temporal fluctuations. Yet, the impact of such pH fluctuations on nasal microbiota is not fully understood. Here, we examine how temporal fluctuations in pH might affect the coexistence of nasal bacteria in *in silico* communities. We take advantage of the cultivability of nasal bacteria to experimentally assess their responses to pH and the presence of other species. Based on experimentally observed responses, we formulate a mathematical model to numerically investigate the impact of temporal pH fluctuations on species coexistence. We assemble *in silico* nasal communities using up to 20 strains that resemble the isolates that we have experimentally characterized. We then subject these *in silico* communities to pH fluctuations and assess how the community composition and coexistence is impacted. Using this model, we then simulate pH fluctuations—varying in amplitude or frequency—to identify conditions that best support species coexistence. We find that the composition of nasal communities is generally robust against pH fluctuations within the expected range of amplitudes and frequencies. Our results also show that cooperative communities and communities with lower niche overlap have significantly lower composition deviations when exposed to temporal pH fluctuations. Overall, our data suggest that nasal microbiota could be robust against environmental fluctuations.

## Introduction

Resident microbes in the human nasal passage protect us from respiratory pathogens (Brugger et al., 2016; Man et al., 2017). Indeed, previous research shows the role of resident commensals in suppressing pathogens, such as *Staphylococcus aureus* (Uehara et al., 2000; Iwase et al., 2010; Bomar et al., 2016). Investigating how this microbial community is formed and maintained can therefore provide powerful insights into microbiota-based therapies to prevent or treat infections. While such an investigation appears formidable in complex environments such as the gut microbiota, it is feasible for nasal microbiota. First, the nasal microbiota has relatively low diversity, with the majority of composition often attributed to 3∼8 species (Escapa et al., 2018). Second, the majority of these species are readily culturable aerobically *in vitro* under controlled environments (Kaspar et al., 2016; Escapa et al., 2018). Third, both the species and the nasal environment can be sampled relatively easily (Yan et al., 2013; Proctor and Relman, 2017). The combination of these factors makes the nasal microbiota a suitable choice for mechanistic studies of human microbiota and a gateway for more detailed studies of human-associated microbiota. Despite these advantages, community-level modeling of nasal microbiota has not been discussed adequately so far. A majority of existing work has focused on the biology of specific members of the nasal microbiota such as *Staphylococcus aureus* or *Streptococcus pneumoniae* because of their disease relevance (Regev-Yochay et al., 2004; Wertheim et al., 2004; Cespedes et al., 2005). Other reports have characterized and investigated the interactions among nasal microbes (Iwase et al., 2010; Bomar et al., 2016), but often with a focus on the interaction itself, and have only rarely involved the ecological consequences for the community (see Margolis et al., 2010; Yan et al., 2013; Krismer et al., 2017, for example).

Many factors, including interspecies interactions (Bomar et al., 2016; Brugger et al., 2016, 2020), the host immune system (Johannessen et al., 2012), and resource availability and access (Relman, 2012) can impact the nasal microbiota. However, all these factors take place in an environment that may fluctuate over time and vary in space. Previous investigations have revealed that the nasal passage is in fact very heterogeneous, both spatially and temporally (Proctor and Relman, 2017). In particular, pH fluctuations (in the range of 5.8–7.2, depending on the sampling site and time) were observed within the nasal passage (Washington et al., 2000; Hehar et al., 2001). Previous studies also demonstrate that temporal environmental fluctuations can transition the community to a different state (Abreu et al., 2020) or increase and support biodiversity (Eddison and Ollason, 1978; Grover, 1988; Abrams and Holt, 2002; Jiang and Morin, 2007; Kremer and Klausmeier, 2013). The explanation is often based on the temporal niche partitioning mechanism; i.e., environment variations creates additional niches and allow for more species to coexist (Chesson, 2000; Amarasekare, 2003). The purpose of our work is not to introduce a new theoretical framework for modeling microbial communities. Instead, we aim for a predictive mathematical model to study the impact of temporal pH fluctuations on the nasal microbiota composition. Other factors notwithstanding, we specifically ask whether, and when, incorporating temporal pH fluctuations is necessary to accurately predict compositional outcomes.

**FIGURE 1:**
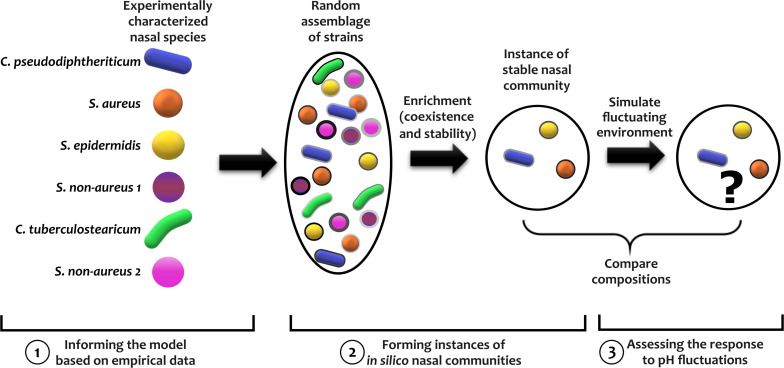
The outline of the procedure for assessing the impact of temporal pH fluctuations on nasal microbiota is shown. To assemble *in silico* nasal communities, we characterized 6 bacterial nasal isolates. We then created *in silico* strains by randomly modulating the parameters of each characterized strain—schematically illustrated as different shades for each species. Using random assemblies of such strains, we simulated the enrichment process to find instances of stable nasal communities. We exposed these communities to a fluctuating pH and compared how the community composition was affected.

To answer the above question, we first characterize six nasal bacterial isolates as representative of members present in the nasal community. The rationale behind choosing these nasal bacteria was that (1) we can culture these strains reliably in the same cultivation medium and conditions in the lab; (2) covering different *Corynebacterium* and *Staphylococcus* species, these strains capture some of the natural diversity of microbiota (Escapa et al., 2018); and (3) Some interactions among these strains has already been identified (Brugger et al., 2020). For instance, *Corynebacterium* have been used to inhibit *S. aureus* colonization (Uehara et al., 2000; Kiryukhina et al., 2013) and *S. aureus* promotes the growth of *C. accolens* and gets inhibited by *C. pseudodiphtheriticum* (Yan et al., 2013). We then use *in vitro* communities constructed from nasal isolates to quantify the community response to temporal pH variations. Then, with parameters relevant to nasal microbiota, we use a phenomenological model to represent microbes and their interactions in an environment with a temporally fluctuating pH. Based on our empirical characterizations of nasal bacteria, we construct *in silico* examples of nasal microbiota and quantify their response to temporal pH fluctuations. Our simulation results suggest that temporal pH fluctuations do not have a major impact on the stable coexistence of nasal bacteria. The outline of our procedure to assess the impact of temporal pH fluctuations on nasal microbiota is shown in Figure 1.

## Materials and Methods

### Nasal Bacterial Strains

Six strains used in this study were isolated from two healthy individuals and kindly shared with us by Dr. Katherine Lemon (Table 1). Interactions between some of these strains and other nasal bacteria has been studied recently (Brugger et al., 2020).

**TABLE 1:** Nasal strains used in this study are listed along with their designation based on 16S rRNA gene similarity.

**Strain name**	**Genus**	**Most likely species designation**
KPL1821	*Corynebacterium*	*Corynebacterium tuberculostearicum*
KPL1828	*Staphylococcus*	*Staphylococcus aureus*
KPL1839	*Staphylococcus*	*Staphylococcus epidermidis*
KPL1850	*Staphylococcus*	*Staphylococcus non-aureus 1*
KPL1989	*Corynebacterium*	*Corynebacterium pseudodiphtheriticum*
KPL1867	*Staphylococcus*	*Staphylococcus non-aureus 2*

**Table:** 

**Parameter**	**Description**	**Value**
*N*_c_	Maximum number of strains for *in silico* community assembly	20
*N*_s_	Number of instances of assembly simulations run for each case	10,000
*N*_gen_	Number of generations simulated to obtain stable resident communities; also the number of generations simulated to assess response to environmental fluctuations	100
*pH*_rng_	Range of pH values (both in experiments and in simulations)	5.1–7.5
δ	Dilution rate	0.03–0.3 h^–1^
*N*_ext_	Extinction population density per species (OD)	10^–6^
*f*_p_	Inter-strain parameter variation within each species	20%
*S*_0_	Average initial cell density per strain (OD)	10^–4^
*f*_pH_	Frequency of sinusoidal temporal pH fluctuations	1 h^–1^
ΔpH	Amplitude of sinusoidal temporal pH fluctuations	0.5

### Cultivation Conditions and Medium *in vitro*

As growth medium, we have used a 10-fold dilution of the Todd-Hewitt broth with yeast extract (THY, at an initial pH of 7.2). We have diluted THY to create an environment closer to the nutrient richness of the nasal passage (Krismer et al., 2014). For collecting cell-free filtrates, cells were grown in 15 ml of media in sterile 50 ml Falcon tubes with loose caps exposed to the room atmosphere. For growth rate and carrying capacity characterizations, cells were grown in flat-bottom 96-well plates. All cultures were grown at 37°C with continuous shaking at 250 rpm.

### Characterizing the pH Response of Nasal Isolates *in vitro*

To assess the response of nasal strains, we grew them in 10% THY after adjusting the pH within the biologically relevant range of 5.1 and 7.5 at 0.3 intervals (pH buffered with 10 g/l of MOPS). For each strain, we measured the growth rate at low population sizes (before nutrients become limiting or byproducts become inhibitory) and the final carrying capacity. These values were measured by growing replicates of each strain (typically 6 replicates) in 96-well microtiter plates incubated inside a Synergy Mx plate reader. Growth rate and carrying capacity were estimated by measuring the absorption in each well (OD_600_) at 10 min intervals over 24 h at 37°C. Between absorption reads, the plate was kept shaking to ensure a well-mixed environment.

### Mathematical Model

To model the growth of species, we assume that in the absence of interactions, the population growth follows the logistic equations:

$$\frac{d\,S_{i}}{d\,t}=r_{i}\,\left(p\right)\,\left[1-\frac{S_{i}}{K_{i}\,\left(p\right)}\right]\,S_{i}-\delta \,S_{i}.$$

In which *r*_*i*_(*p*) and *K*_*i*_(*p*) are the pH-dependent growth rate and carrying capacity of species *i*. In our simulations, the growth rate and carrying capacity values at any given pH are found using a linear interpolation from experimentally measured values (pH 5.1–7.5 at 0.3 intervals). pH dependence is experimentally characterized for each strain in a monoculture, as described above, and δ is the dilution rate.

When multiple species are present, we assume that the presence of other species takes away resources from the environment; as a result, the growth of each species will be modulated as

$$\frac{d\,S_{i}}{d\,t}=r_{i}\,\left(p\right)\,\left[1-\frac{S_{i}-\gamma_{i}}{K_{i}\,\left(p\right)}\right]\,S_{i}-\delta \,S_{i}.$$

where $\gamma_{i}=\sum_{j\neq i}c_{i\,j}\,S_{j}$ and *r*_*i*_(*p*)*c*_*ij*_/*K*_*i*_(*p*) represents the interaction strength exerted on species i by species j. Positive values of *c*_*ij*_ indicate growth stimulation (e.g., via facilitation by producing resources) whereas negative values of *c*_*ij*_ indicate growth inhibition (e.g., via competition).

### Model Parameters

Unless otherwise specified, the following parameters are used in the model:

Some of these parameters, such as the range, frequency, and amplitude of pH values are chosen to keep the simulations close to what is expected in the nasal environment (Washington et al., 2000; Hehar et al., 2001). Some of the other parameters, such as the dilution rate or the initial and extinction population densities are not expected to be critical for the overall conclusions of this work. We have chosen these parameters to reflect realistic parameters that can be later tested experimentally. Finally, parameters such as the number of instances simulated (*N*_s_) and the number of generations simulated (*N*_gen_) are chosen to give us enough confidence for our claims, while keeping the practical considerations of simulation time and effort in mind.

### Characterizing the Interspecies Interactions Using a Supernatant Assay

To characterize how species *j* affects the growth of other species *i*, we use a supernatant assay in which species *j* is grown to saturation, then all the cells are filtered out using a 0.22 μm filter (PVDF syringe filters from Thomas Scientific). The growth rate and carrying capacity of species *i* is then measured when growing in the supernatant taken from cultures of species *j*. This formulation allows us to use the experimentally measurable supernatant responses to formulate a dynamical model for mixed cultures of multiple species.

Assuming a Lotka-Volterra model, the presence of another species modulates the growth rate proportionally to the size of the interacting partner, i.e.,

$$\frac{d\,S_{i}}{d\,t}=r_{i}\,\left[1-\frac{S_{i}-c_{i\,j}\,S_{j}}{K_{i}}\right]\,\left(1-\frac{S_{i}-c_{i\,j}\,S_{j}}{K_{i}}\right)\,S_{i}.$$

Calculating the parameters obtained from the cell-free spent media (CFSM), the carrying capacity for species *i* is reached at population *S*_*i,cc*_ level when growth rate becomes zero, thus

$$\left(1-\frac{S_{i,c\,c}-c_{i\,j}\,K_{j}}{K_{i}}\right)=0.$$

Therefore, the carrying capacity in the supernatant assay (*K*_*ij*_) is

$$K_{i\,j}=S_{i,c\,c}=K_{i}+c_{i\,j}\,K_{j}.$$

And the interaction coefficient (*c*_*ij*_, effect of species *j* on species *i*) can be calculated as

$$c_{i\,j}=\frac{K_{i\,j}-K_{i}}{K_{j}}.$$

In the particular that species *i* and *j* are similar (self-effect), we have *K*_*ii*_ = 0 and *c*_*ii*_ = −1. It should be noted that there are limitations in using a Lotka-Volterra model. Such models may not accurately represent microbial interactions (Momeni et al., 2017). Additionally, under certain conditions, the solutions will exhibit instability. Particularly for this latter case, we examine the situations under which “runaway” growth instability may happen. Consider two mutualistic populations:

$$\frac{d\,S_{1}}{d\,t}=r_{1}\,\left[1-\frac{S_{1}-c_{12}\,S_{2}}{K_{1}}\right]\,S_{1}\,\text{and}\,\frac{d\,S_{2}}{d\,t}=r_{2}\,\left[1-\frac{S_{2}-c_{21}\,S_{1}}{K_{2}}\right]\,S_{2}$$

Instability can happen when the carrying capacity terms fail to act as a negative feedback to bound the population. This can happen when

$$\left[1-\frac{S_{1}-c_{12}\,S_{2}}{K_{1}}\right]>1\,\text{and}\,\left[1-\frac{S_{2}-c_{21}\,S_{1}}{K_{2}}\right]>1$$

This happens when *S*_1_ < *c*_12_*S*_2_ and *S*_2_ < *c*_21_*S*_1_. Satisfying both of these inequalities requires that *c*_*12*_ and *c*_12_*c*_21_ > 1, which means strong mutual facilitation. In our dataset, we do not have examples of mutual or cyclic facilitation and facilitation interaction terms are small, suggesting that instability is not expected in our simulations. Nevertheless, these conditions should be kept in mind for other datasets, especially those with strong facilitation between community members.

### Calculating Community Composition Deviations

To compare community composition of a community that experienced pH fluctuation with that of the same community simulated at a fixed pH, we calculated the Bray-Curtis dissimilarity measure using the f_dis function (option “BC”) in MATLAB. The necessary files to reproduce the analysis are included in the accompanied source codes^1^.

### Estimating the Impact of pH Fluctuations

We consider two extremes, when the fluctuations in pH are (1) much faster or (2) much slower than the population dynamics of community members. In both cases, for our formulation we define *c*_*ii*_ = −1 and use the simplified model of populations at different pH:

$$\frac{d\,S_{i}}{d\,t}=r_{i}\,\left(p\right)\,\left[1+\frac{\hat{\gamma_{i}}}{K_{i}\,\left(p\right)}\right]\,S_{i}-\delta \,S_{i}$$

and

$$\hat{\gamma_{i}}=\sum c_{i\,j}\,S_{j}$$

Case 1. Fast pH fluctuations: To estimate how the community responds under a rapidly changing pH, we use the framework of the Wentzel–Kramers–Brillouin (WKB) approximation. For the general case of $\frac{d\,S}{d\,t}=r\,\left(t\right)\,S$, we split the population dynamics into two terms, the primary exponential term and an envelope function, *E*, for which *E*(*t*) = *e*^−*r*_0_*t*^*S*(*t*) and thus,

$$\frac{d\,E}{d\,t}=\left[r\,\left(t\right)-r_{0}\right]\,E$$

Using the WKB approximation *E* can be written using the expansion

$$E=\text{exp}\,\left[\frac{1}{\varepsilon }\,\sum_{n=0}^{\infty }\varepsilon^{n}\,E_{n}\,\left(t\right)\right]$$

By inserting this expansion into the differential equation, we obtain

$$\left(\frac{1}{\varepsilon }\,\sum_{n=0}^{\infty }\varepsilon^{n}\,\frac{d}{d\,t}\,E_{n}\,\left(t\right)\right)\,\text{exp}\,\left[\frac{1}{\varepsilon }\,\sum_{n=0}^{\infty }\varepsilon^{n}\,E_{n}\,\left(t\right)\right]=\left(r\,\left(t\right)-r_{0}\right)\,\text{exp}\,\left[\frac{1}{\varepsilon }\,\sum_{n=0}^{\infty }\varepsilon^{n}\,E_{n}\,\left(t\right)\right]$$

Thus

$$\frac{1}{\varepsilon }\,\sum_{n=0}^{\infty }\varepsilon^{n}\,\frac{d}{d\,t}\,E_{n}\,\left(t\right)=r\,\left(t\right)-r_{0}$$

Assuming sinusoidal changes in pH, *p*(*t*) = *p*_0_ + *p*_*d*_sin(2π*ft*), to the first order, the temporal changes in growth rate can be approximated as, *r*(*t*) = *r*_0_ + *r*_*d*_sin(2π*ft*). Therefore,

$$\frac{1}{\varepsilon }\,\sum_{n=0}^{\infty }\varepsilon^{n}\,\frac{d}{d\,t}\,E_{n}\,\left(t\right)\approx r_{d}\,\sin\left(2\,\pi \,f\,t\right).$$

In the limit that ε→0the first terms of expansion for *E* are obtained as

$$\frac{d}{d\,t}\,E_{0}\,\left(t\right)=0,.$$

$$\frac{d}{d\,t}\,E_{1}\,\left(t\right)\approx r_{d}\,\sin\left(2\,\pi \,f\,t\right)$$

Since the continuous dilutions in our setup keeps the populations finite, *E*_*0*_ does not affect the solution. The dominant term for *E* thus becomes *E*_1_ and we have

$$E_{1}\,\left(t\right)\approx \frac{-r_{d}}{2\,\pi \,f}\,\cos\left(2\,\pi \,f\,t\right)$$

Aa a result,

$$E\,\left(t\right)\propto \text{exp}\,\left[\frac{-r_{d}}{2\,\pi \,f}\,\cos\left(2\,\pi \,f\,t\right)\right]$$

Importantly, the magnitude of change in this equation drops inversely proportional to the frequency of pH fluctuations *f*. This means that the impact of pH fluctuations diminishes at high frequencies, consistent with our intuition that in this case the community dynamics are incapable of following the environmental fluctuations and only respond to the mean value.

Case 2. Slow pH fluctuations: In this case, we assume the quasi-static approximation, in which fluctuations are so slow that the community approaches its steady-state at each temporal value of pH. In this situation, assuming $\frac{d\,S_{i}}{d\,t}=0,$ we can rearrange the equation at steady-state as

$$\left[r_{i}\,\left(p\right)-\delta \right]\,S_{i}=-\frac{r_{i}\,\left(p\right)}{K_{i}\,\left(p\right)}\,S_{i}\,\sum c_{i\,j}\,S_{j}$$

Rearranging this, we get

$$\left[\delta -r_{i}\,\left(p\right)\right]\,\frac{K_{i}\,\left(p\right)}{r_{i}\,\left(p\right)}=\sum c_{i\,j}\,S_{j}.$$

This can be written in matrix form as

$$\left[C\right]\,\underline{S}=\underline{b},$$

where [*C*] contains the interaction coefficients and $b_{i}=\left[\delta -r_{i}\,\left(p\right)\right]\,\frac{K_{i}\,\left(p\right)}{r_{i}\,\left(p\right)}$; underline in our notation designates a vector. Since the interaction matrix [*C*] is pH-independent in our model, the change in composition within this quasi-static approximation can be expressed as

$$\left[C\right]\,\left(\underline{S\,\left(t\right)}-\underline{S_{0}}\right)=\underline{b\,\left(t\right)}-\underline{b_{0}},$$

or.

$$\underline{\Delta \,S\,\left(t\right)}=\left(\underline{S\,\left(t\right)}-\underline{S_{0}}\right)={\left[C\right]}^{-1}\,\left(\underline{b\,\left(t\right)}-\underline{b_{0}}\right)={\left[C\right]}^{-1}\,\underline{\Delta \,b\,\left(t\right)}$$

We make an additional simplifying assumption that *K*_*i*_ and *r*_*i*_ change similarly with pH. This leads to $\Delta \,b_{i}\approx \left(r_{i\,0}-r_{i}\,\left(t\right)\right)\,\frac{K_{i\,0}}{r_{i\,0}}$. This means that the magnitude of change in community composition is the same as the change in the growth rate of species, regardless of the frequency of fluctuations, under this regime.

### Allowing pH-Dependent Interaction Coefficients

To examine how pH-dependent interaction coefficients may affect our results, we assumed that each interaction coefficient has a linear dependence on pH with a slope (per unit pH) randomly selected from a uniform distribution in the range of [−*m*, *m*]. In other words,

$$c_{i\,j}\,\left(p\right)=c_{i\,j}\,\left(p_{0}\right)+m\,\left(p-p_{0}\right),$$

where *p*_*0*_ = 7.2 is the pH at which our characterization is performed. We examined how the community composition deviated from the reference with a fixed pH, as *m* (and thus the pH-dependency) increased.

## Results

### *In vitro* Characterization of Nasal Bacteria

We experimentally characterized how six representative nasal bacterial strains respond to different pH values in their environment. These bacterial strains were chosen from a set of isolates (see section “Materials and Methods”) based on three major considerations: (1) they reliably grow in our cultivation media under an aerobic environment; (2) they include commonly observed *Staphylococcus* and *Corynebacterium* species; and (3) they span the phylogenetic landscape of both closely and distantly related bacteria found in the nasal environment (Escapa et al., 2018). We assumed that each of these characterized strains is a representative strain of the corresponding species.

**FIGURE 2:**
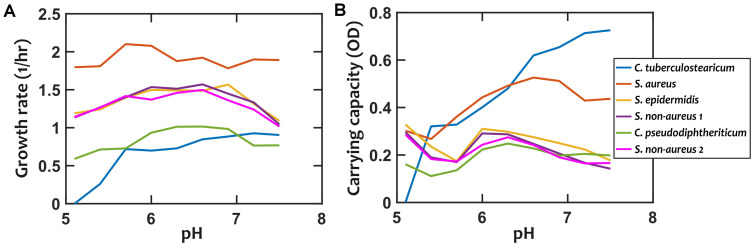
Growth properties of nasal bacterial isolates are pH-dependent. Growth is characterized using the growth rate in the early exponential phase **(A)**, and the carrying capacity based on optical density (OD, absorption measured at 600 nm) as a proxy **(B)**. Each data point is the average of at least 6 replicates from two independent experiments. Error-bars are not shown to avoid overcrowding the plot but the values are available in the raw data. In all cases, growth is experimentally tested in a 10-fold diluted Todd-Hewitt broth with yeast extract (10% THY).

**FIGURE 3:**
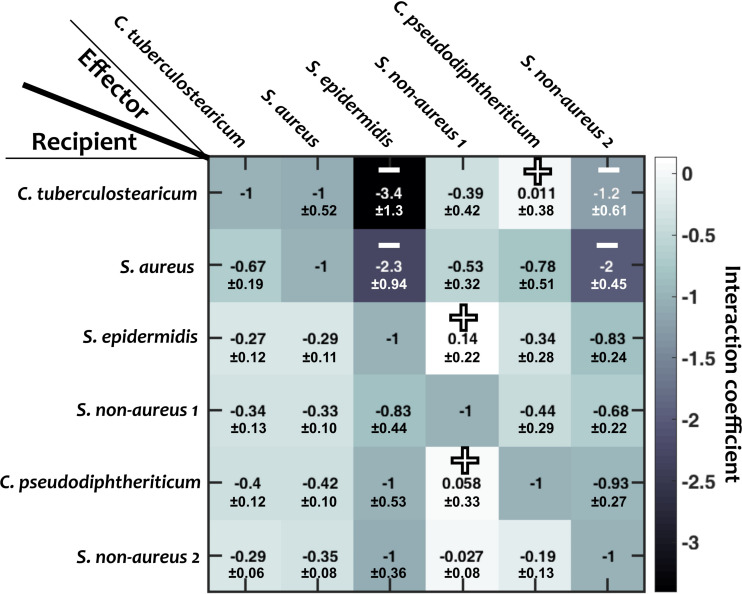
Interaction coefficients among pairs of nasal bacteria. Values represent interaction coefficients in a Lotka-Volterra model. In each case, the growth of a recipient strain is measured when the strain is exposed to cell-free filtrate derived from the effector strain. Positive mean coefficients (indicating facilitation) and negative mean coefficients below –1.2 (indicating strong inhibition) across different replicates are marked by “+” and “–,” respectively. Standard deviations (shown for each value) are calculated based on empirical standard deviations of measured carrying capacities in monocultures and supernatant experiments. Diagonal elements are set to –1, indicating complete niche overlap.

We first characterized the pH response of each strain by growing them under different environmental pH values. Different strains exhibited different degrees of pH dependency in their growth rates and carrying capacities (Figure 2 and Supplementary Figures S1, S2). Among these strains, *S. epidermidis*, *S. non-aureus 1*, and *S. non-aureus 2* show fairly similar growth properties. We chose to treat these as separate species in our investigation, because—as shown later—they had considerably different interactions with other species (Figure 3).

We then examined how different species interact with one another. For this, we grew each species to its stationary phase in a monoculture, filtered out the cells, and measured how other strains grew in the resulting cell-free filtrates (see section “Materials and Methods”; similar to De Vos et al., 2017; Brugger et al., 2020). From these measurements, we estimated the inter-species interaction coefficients based on the generalized Lotka-Volterra model (Figure 3; see section “Materials and Methods”). In this formulation, baseline competition with complete niche overlap will result in an interaction coefficient of −1. Of note, from our experimental data we cannot distinguish the relative contribution of competitive niche overlap and interspecies facilitation. Nevertheless, for simplicity we only use “facilitation” for extreme cases in which facilitation outweighs competition and the interaction coefficient turns positive. We interpret different gradations of negative interaction coefficients from −1 to 0 as different degrees of niche overlap (with −1 indicating complete niche overlap), and cases with interaction coefficients less than −1 indicate inhibition beyond competition for resources. Among the 30 pairwise interaction coefficients, there were 3 positive values (bright blue, marked by “+” in Figure 3). For simplicity, throughout this manuscript, we assume that these interaction coefficients are not pH-dependent.

### *In silico* Assembly of Nasal Bacterial Communities

To capture some of the diversity of nasal microbiota, we propose that other *in silico* strains of each species can be constructed by randomly modulating the measured properties of that species (i.e., growth rate, carrying capacity, and interaction coefficients). We chose the degree of strain-level modulation to be up to 20%, as a balance between intraspecies and interspecies diversity (Supplementary Figure S3).

To assess the response of nasal microbiota to temporal fluctuations in the environment, we first construct an ensemble of *in silico* communities that represent a subset of possible nasal communities. This is chosen as an alternative to performing an *in vivo* study, because performing these experiments with human subjects is not feasible and there is no reliable animal model for human nasal bacteria. Our approach is, in essence, similar to several other previous work that have used simple models to describe the dynamics of human-associated microbiota (Stein et al., 2013; Fisher and Mehta, 2014; Song et al., 2014; De Vos et al., 2017; Venturelli et al., 2018). Compared to *in vitro* studies, these *in silico* communities give us full control over confounding factors and allows us to examine the mechanisms contributing to sensitivity to pH fluctuations (Momeni et al., 2011). To construct *in silico* communities, we mimicked enrichment experiments (Goldford et al., 2018; Niehaus et al., 2019) by simulating the dynamics of an initial assemblage of 20 strains (sampled from the space of *in silico* strains) until the community reached stable coexistence. These *in silico* communities were largely robust against experimental noise in characterization (Supplementary Figure S4). The interspecies interactions in our model appear to be instrumental in the assembly of these *in silico* communities, as evidenced by changes when we assigned the interaction coefficients at given levels (Supplementary Figure S5A) or modulated the measured interactions (Supplementary Figure S5B). To assess how pH fluctuations in the environment influence nasal communities, we take several instances of *in silico* nasal communities, expose them to a fluctuating pH, and quantify how the community composition is affected. The entire process is outlined in Figure 1.

### *In silico* Nasal Communities Are Diverse and Favor Facilitation

We first examined the properties of assembled *in silico* communities at various pH values with no temporal fluctuations. We found that the prevalence of different species was distinct and pH-dependent (Supplementary Figure S6). This prevalence is a result of nasal species’ pH-dependent growth properties as well as their interspecies interactions.

We also found that during the process of assembling *in silico* communities, the prevalence of interspecies facilitation interactions increased. Comparing the prevalence of facilitation in initial assemblages of strains vs. the final stable communities, we found that among the communities that had at least one facilitation interaction at the start of the *in silico* enrichment (89% of communities), facilitation was enriched in ∼66% of the final community assemblies (Supplementary Figure S7).

### Temporal pH Fluctuations Only Minimally Impact Nasal Microbiota Composition

Next, we asked how the temporal variation in the environment might influence the community composition. To answer this question, we used instances of *in silico* communities to evaluate the impact of temporal pH fluctuations. We assumed a continuous growth situation in which all community members experience a constant dilution rate. This dilution mimics the turnover in microbiota, for example, when the mucosal layer gets washed away. To avoid situations in which the *in silico* community itself was not stable, we changed the dilution rate by ± 50% and only kept the communities for which the modified dilutions only caused a small deviation in community composition (see section “Materials and Methods”). Indeed, we found that communities with compositions more sensitive to dilution rates are also more sensitive to pH fluctuations (Supplementary Figure S8). In all cases, composition deviations were calculated using the Bray-Curtis dissimilarity measure (see section “Materials and Methods”).

**FIGURE 4:**
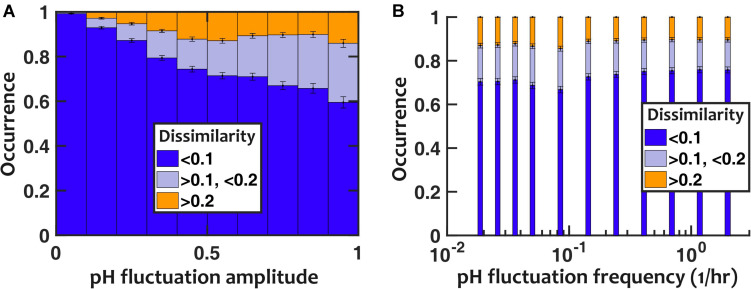
Nasal microbiota composition is robust against pH fluctuations. **(A)** For a fixed fluctuation frequency (*f*_pH_ = 0.2/h), larger fluctuation amplitudes increase how the community composition deviates from the no-fluctuation steady state (as quantified with composition dissimilarity). **(B)** The impact of temporal fluctuations is maximum at intermediate frequencies. Here, the pH fluctuation amplitude is fixed (ΔpH = 0.5). Number of *in silico* communities examined for each condition: *n* = 10,000.

To evaluate the impact of pH fluctuations, we simulated a controlled sinusoidal pH variation over time, with two parameters: the amplitude and frequency of temporal variations. Thus, *p*(*t*) = *p*_0_ + Δ*pHsin*(2π*f*_pH_*t*). Keeping the frequency of fluctuations fixed (*f*_pH_ = 0.2/h), we observed that the deviation in population composition increased with an increasing pH fluctuation amplitude (ΔpH). However, the resulting dissimilarity in population composition was mostly minor, with > 85% of cases showing less than 0.2 dissimilarity even when the amplitude of pH fluctuation was set to 1 (Figure 4A). We then examined the impact of the frequency of pH variations, while we kept the amplitude of pH fluctuations fixed (ΔpH = 0.5). At intermediate frequencies, the pH fluctuations caused the largest dissimilarity in community composition compared to stable communities with fixed pH (Figure 4B).

We repeated the assessment of pH fluctuations by assuming a pH that randomly fluctuated between two discrete pH values to ensure that our results were not limited to sinusoidal fluctuations. The results were overall consistent with sinusoidal pH fluctuations (Supplementary Figure S9): (1) larger pH fluctuation amplitudes increased the deviation in population composition, but overall the majority of communities only experienced modest deviations; and (2) pH fluctuations at intermediate frequencies had the largest impact on community composition.

### Interspecies Facilitations Dampen the Impact of Temporal Fluctuations

**FIGURE 5:**
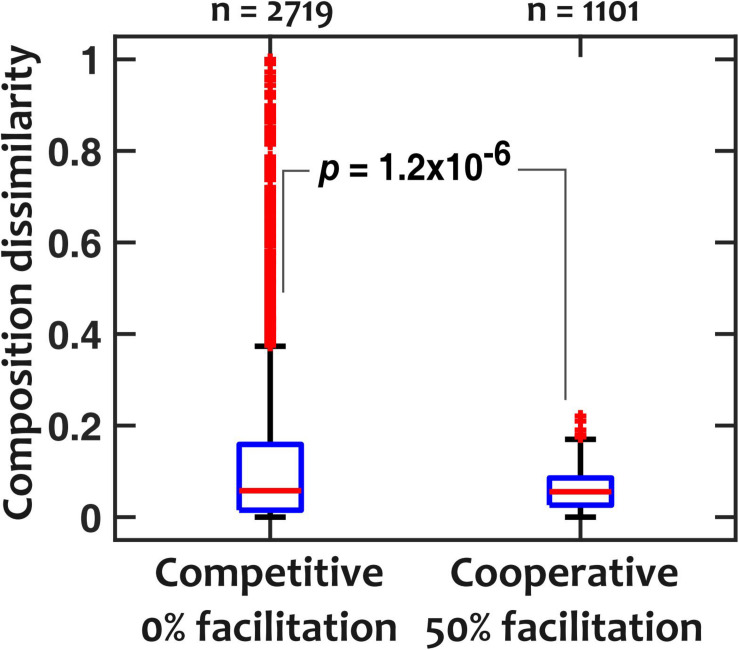
Cooperative communities are more robust against pH fluctuations compared to competitive communities. For competitive (those with 0% facilitation among members) and cooperative (those with 50% facilitation interactions among members) communities, dissimilarity medians were 0.057 and 0.055 and dissimilarity means were 0.12 and 0.058, respectively (*p* = 1.2 × 10^– 6^ with a Mann-Whitney *U*-test). pH fluctuates sinusoidally with a frequency of *f*_pH_ = 0.2/h and an amplitude of ΔpH = 0.5.

To explain the low sensitivity of community composition to pH fluctuations, we hypothesized that interspecies facilitation stabilizes the composition by creating interdependencies within the community. From the data in Figure 4, we picked and compared communities with low (“competitive,” 0% facilitation) and high (“cooperative,” 50% facilitation) prevalence of facilitation. The 0% facilitation corresponds to situations where none of the strains facilitate any of the other members of the *in silico* community. In contrast, 50% facilitation happens when half of the pairwise interactions among the *in silico* community are facilitative. Since in our dataset (Figure 3) there were no instances of mutual facilitation, 50% facilitation is the maximum fraction that cooperative communities can reach. The results show that cooperative communities have a consistently and significantly lower composition deviation when exposed to temporal pH fluctuations (Figure 5).

**FIGURE 6:**
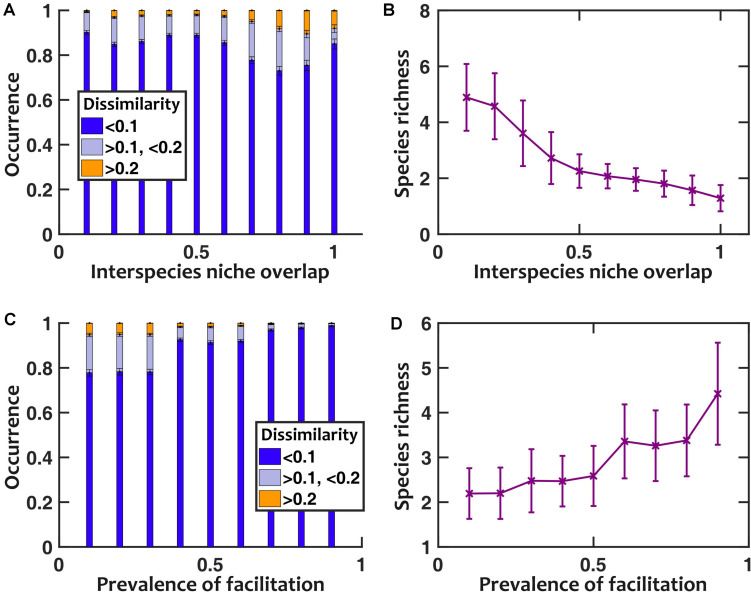
Lower niche overlap and more prevalent facilitation decrease the sensitivity to pH fluctuations. **(A)** As we artificially increased the strength of niche overlap (by setting all off-diagonal coefficients in the interaction matrix to be a fixed negative number and making that number more negative), the sensitivity to pH fluctuations increased. This trend is disrupted when niche overlap approaches 1, because a majority of communities under such conditions lose interspecies diversity **(B)**. As we increased the prevalence of interspecies facilitation by randomly setting a given fraction of interaction coefficients to be positive, the communities became less sensitive to pH fluctuations **(C)** and more diverse in species richness **(D)**. Here pH fluctuations are sinusoidal, with *f*_pH_ = 0.2/h and ΔpH = 0.5.

To further explore the impact of facilitation, we asked how interspecies niche overlap (the magnitude of negative interspecies interactions) and prevalence of facilitation (the fraction of interspecies interactions that are positive) contribute to sensitivity to pH fluctuations. In our results, we found that larger interspecies niche overlap leads to more sensitivity to pH fluctuations (Figure 6A). This trend holds except when interspecies niche overlap approaches 1; at such high overlaps the community loses diversity (Figure 6B), becoming less sensitive to pH fluctuations. When we directly changed the prevalence of facilitation, we observed that with higher prevalence of facilitation the communities became more diverse and less sensitive to pH fluctuations (Figures 6C,D).

## Discussion

Using empirically measured species properties, we assembled stable *in silico* communities that show coexistence of nasal bacteria. When these communities were exposed to a fluctuating pH environment, we observed that the composition of stable communities was only modestly affected. Larger pH fluctuations increased the deviation, as expected; however, even at a pH fluctuation of 1 which exceeds the observed temporal variation in the nasal passage, the composition of the majority of communities remained minimally affected. We also found that intermediate frequencies of temporal pH fluctuations caused the largest deviations in community compositions. Finally, in our results, communities with more facilitation interactions were more robust against pH fluctuations.

In choosing an appropriate model, one must carefully consider the processes of interest and the required level of abstraction to capture those processes (Momeni et al., 2011; Silverman et al., 2018b). Models originally designed for single species populations have been adapted to characterize microbial communities. Community ecology modeling frameworks designed to understand interactions at the macro scale—in both space and time—have also been applied to microbial populations to study the dynamics of succession and restoration, along with the impact of environmental disturbances (Byrd and Segre, 2016; Gilbert and Lynch, 2019). For example, flux balance analyses, a mainstay in microbial metabolic models, can be modified to describe species interactions within a complex microbial community over time (Larsen et al., 2012; Gerber, 2014; Bucci et al., 2016; Fukuyama et al., 2017; Äijö et al., 2018; Silverman et al., 2018a; Shenhav et al., 2019). To create a predictive model for nasal microbiota, we have extended the generalized Lotka-Volterra (gLV) equations to study the impact of pH fluctuations on community composition. Generalized Lotka-Volterra equations have been previously used to investigate species interactions in the human gut (Fisher and Mehta, 2014; Song et al., 2014) and in a cheese-associated microbial community (Äijö et al., 2018; Song et al., 2014). It has also been similarly extended to describe the impact of environmental fluctuations (antibiotics) on gut microbiota (Stein et al., 2013; Song et al., 2014). For our data, the Lotka-Volterra-type model has proven to—at least to the first-order—capture species growth, interactions, and pH-dependence.

One important aspect of temporal fluctuations is their time scale. Even though in nature the fluctuations are not completely regular, our investigation with sinusoidal temporal fluctuations reveal the time scale at which the influence on community composition is the strongest. Our analysis reveals that the fluctuations are more impactful at intermediate frequencies between two extremes (see section “Materials and Methods”). At very low frequencies of pH fluctuations, the community dynamics are faster than pH changes; thus we can assume the quasi-static approximation applies. In this regime, the community reaches its stable state locally (in time), and the composition follows the value of pH at any given time, regardless of the frequency of the pH fluctuations. In the other extreme, at very high frequencies of pH fluctuations, the population dynamics cannot follow rapid changes in pH and essentially the species “see” the average pH. An analysis based on the Wentzel–Kramers–Brillouin (WKB) approximation suggests that in this regime, the magnitude of change in composition (compared to the composition at the average pH) is inversely proportional to the pH fluctuation frequency. Between these two extremes is the zone that exhibits the most change in community composition with pH fluctuations (Figure 4B and Supplementary Figure S9B). However, for parameters relevant to the nasal strains we are analyzing, even in this zone the changes in community composition are not drastic.

Our focus in this manuscript is on how composition of stable communities changes when environmental pH fluctuates. Another relevant question is how fluctuations in pH affect the process of community assembly. For this, we repeated the community assembly simulations (Supplementary Figures S6,S7), but under an environment in which the pH temporally fluctuated. Contrary to our expectation, the richness of resulting communities did not monotonically increase with an increase in the amplitude of pH fluctuations, regardless of fluctuation frequency (Supplementary Figure S10). Instead, we found that richness only changed in a small fraction of *in silico* nasal communities. Furthermore, in cases with increased richness, *S. non-aureus* 1 (most facilitative species in our panel) was most frequently added to the community, whereas in cases with decreased richness, *S. epidermidis* (most inhibitory species in our panel) was most frequently dropped from the community (Supplementary Figure S11). This observation underscores the relative importance of interaction (compared to niche partitioning) in richness outcomes in our model of nasal communities. Our finding is also consistent with predictions about augmentation and colonization resistance using a mediator-explicit model of interactions (Kurkjian et al., 2020).

There are some limitations and simplifications in our study. First, in our investigation we have assumed that fluctuations in pH are imposed externally (e.g., by the host or the environment). It is also possible that species within the nasal community contribute to the environmental pH. Although outside the scope of this work, we speculate that if species within the community drive the pH to specific values (Ratzke and Gore, 2018; Ratzke et al., 2018), the impact of external temporal fluctuations of pH on community composition will be even more diminished. Second, in our model, we assumed that interactions among species remained unchanged at different environmental pH values. We examined *in silico* how pH-dependent interaction coefficients might affect our results. For this, we assumed that interaction coefficients changed linearly with pH in each case (see section “Materials and Methods”) and asked how strong the dependency had to be to considerably change the community composition under a fluctuating pH. We observed a significant impact only when the interaction coefficients were strongly pH dependent (i.e., to the level that the sign of interactions would change within the range of pH fluctuations) (Supplementary Figure S12).

Our work suggests that a shift in pH can change the community composition and coexistence (Supplementary Figure S6). This is consistent with previous observations from profiling different locations along the nasal passage (Yan et al., 2013). However, our prediction is that temporal pH fluctuations often do not cause a major shift in community structure. As a future step, we plan to verify this prediction experimentally by testing how pH fluctuations affect *in vitro* nasal communities. If confirmed, our prediction is that the spatial position of sampling and the heterogeneity of the environment will have a stronger effect on community composition compared to the temporal resolution of sampling. The practical implication is that microbiome profiling of nasal microbes may not require a high temporal resolution. We proposed that high throughput sampling of the nasal microbiome along with the corresponding pH would be an insightful future step to test our predictions.

Finally, one of the main messages of our work is that nasal microbiota is insensitive to temporal fluctuations in pH. It is tantalizing to speculate, when examining other microbial communities, under what conditions this statement is valid. Recent work by Shibasaki et al. (2020) shows that under a fluctuating environment species properties play an important role in community diversity. Our results corroborate their finding. Insensitivity of the members to the environmental fluctuations—as trivial as it may sound—is a defining factor for how sensitive the community is. In the nasal microbiota, species that we are examining are adapted to the nasal environment and the range of pH fluctuations experienced in this environment is not large. As a result, the community is not majorly affected by pH fluctuations. On top of this, we also observe that interactions—in particular, facilitation and competition—can act as stabilizing or de-stabilizing factors for how the community responds to external variations. In other words, facilitation between community members acts as a composition stabilizing factor between populations, which lowers the impact of external fluctuations (Figure 6A). In contrast, inhibition between community members typically exaggerates the changes introduced by external fluctuations (Figure 6C).

## Data Availability Statement

Codes related to this manuscript can be found at: https://github.com/bmomeni/temporal-fluctuations. The raw data supporting the conclusions of this article will be made available by the authors, without undue reservation.

## Author Contributions

SD, NZ, Y-YL, and BM conceived the research. SD and BM designed the simulations and experiments and wrote the manuscript. SD ran the experiments. SD, MA, SKD, NZ, and BM ran the simulations. SD, MA, SKD, NZ, Y-YL, and BM edited the manuscript. All authors contributed to the article and approved the submitted version.

## Conflict of Interest

The authors declare that the research was conducted in the absence of any commercial or financial relationships that could be construed as a potential conflict of interest.

## Footnotes

1 https://github.com/bmomeni/temporal-fluctuations
