# Supplementary material for: Impact of Temporal pH Fluctuations on the Coexistence of Nasal Bacteria in an in silico Community
Source: Front Microbiol. 2021 Feb 10;12:613109. doi: 10.3389/fmicb.2021.613109 (PMC7902723; doi:10.3389/fmicb.2021.613109)
Supplement: Supplementary file 1 [file Data_Sheet_1.pdf]

Supplementary Material

1 Supplementary Figures

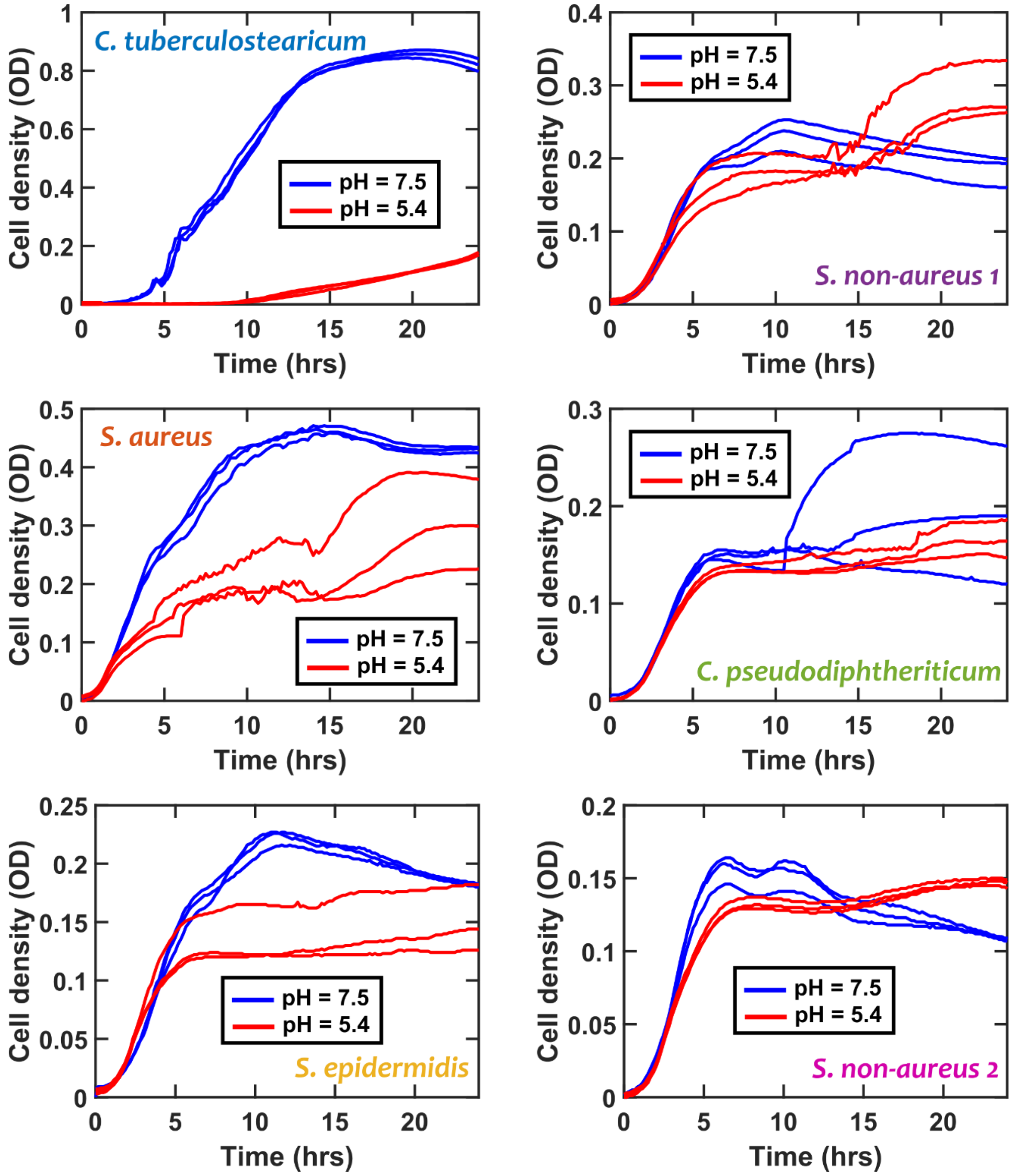

Supplementary Fig S1. Nasal species exhibit different growth properties when grown at different pH values. For two representative pH values (5.4 and 7.5) the growth dynamics of the six nasal species studied in this work are shown (using OD as a proxy). Results are shown for 3 replicates in each case, after subtracting the background OD. The growth conditions match those listed in the Methods section.

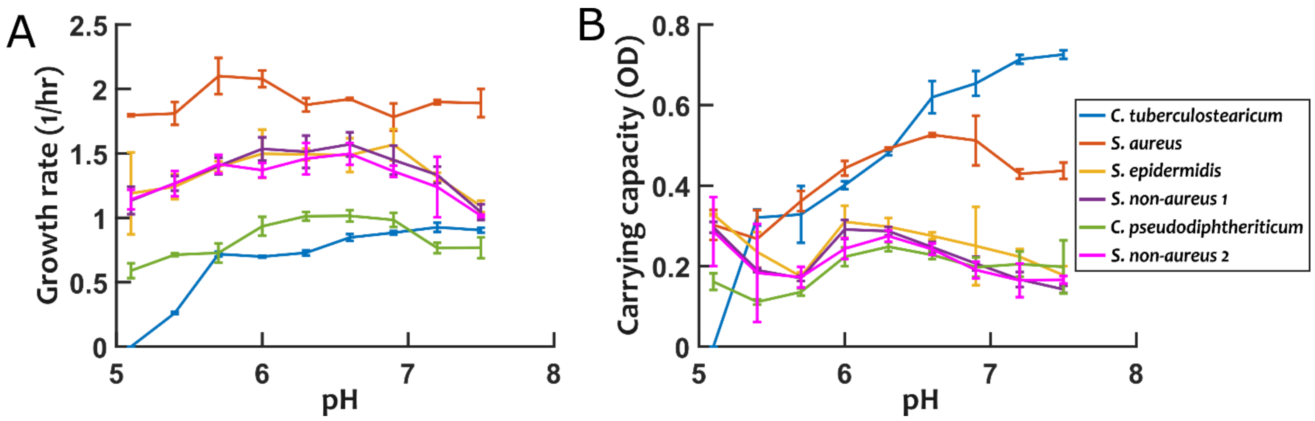

Supplementary Fig S2. Growth properties of nasal bacterial isolates are pH-dependent. Growth is characterized using the growth rate in the early exponential phase (A), and the carrying capacity based on optical density (OD, absorption measured at 600 nm) as a proxy (B). Each data point is the average of at least 6 replicates from two independent experiments. Error-bars show standard deviations among different replicates around the mean value. Growth conditions match those listed in the Methods section.

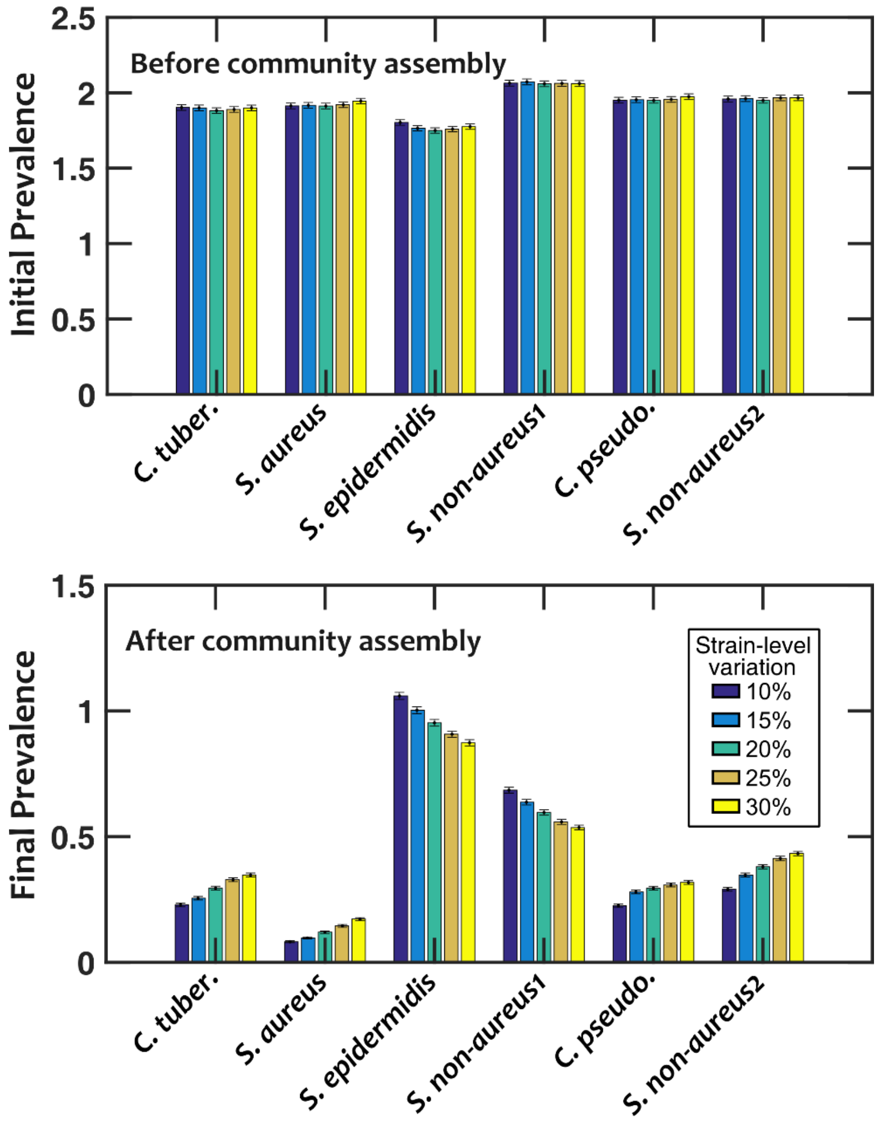

Supplementary Fig S3. The degree of intraspecies parameter variations is chosen as a balance between strain-level and species-level diversity. Starting from communities that had a balanced representation of different strains (top), we observed that after community assembly (bottom), the prevalence of different species was affected. The prevalence here is defined as the average number of *in silico* strains from each species that appeared in each community. The pattern was more pronounced with smaller strain-level variation of 10%, and as this variation increased to 30%, the diversity between different species decreased and species prevalence became more uniform. We chose the strain-level variation of 20% to maintain some strain-level diversity, without diminishing species-level diversity. Number of *in silico* communities examined, n = 10,000.

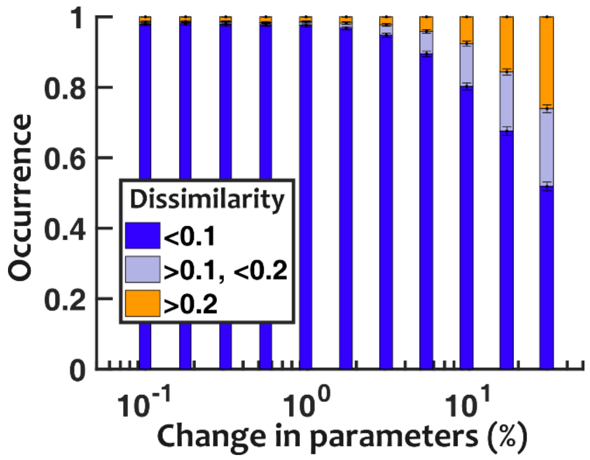

Supplementary Fig S4. Sensitivity analysis shows that *in silico* communities are largely insensitive to measurement noise. To assess whether *in silico* communities were sensitive to experimental error in characterizing species parameters, we intentionally introduced change in measured parameters and quantified the resulting change in community composition. We observe that even when we changed all parameters by up to 10% using a uniform distribution, in silico community compositions were hardly affected. Number of cases examined, n = 10,000.

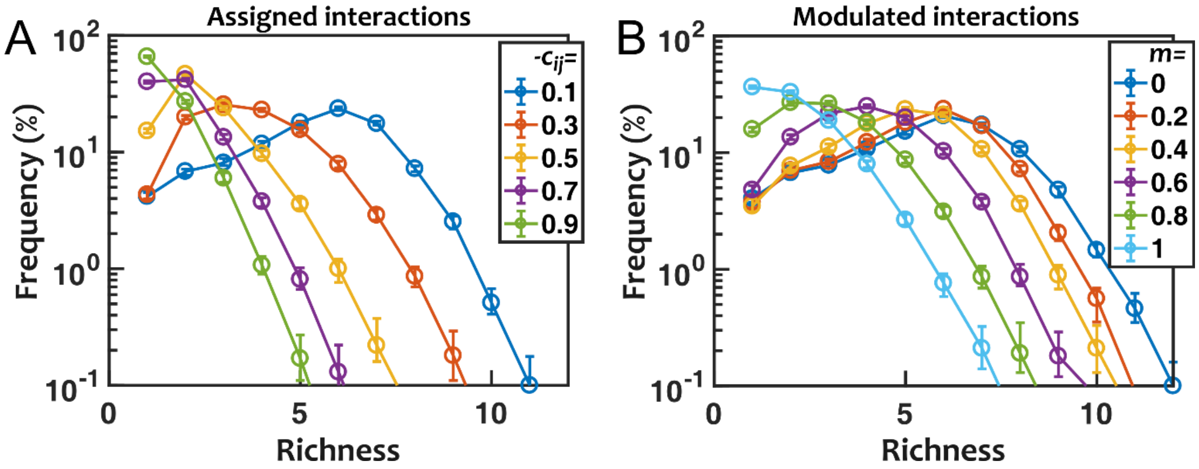

Supplementary Fig S5. Assembly of *in silico* communities is affected by interspecies interactions. To assess the impact of interactions, in our model (A) we assigned all off-diagonal interaction coefficients to a fixed number, c_{ij}, or (B) modulated all experimentally measured off-diagonal interaction coefficients by a fixed factor, m. Both results show that when negative off-diagonal terms (i.e. non-self competitions) are weaker, there is more coexistence. In all cases and for all species, we assumed c_{ii} = -1. Error-bars show bootstrap 95% confidence intervals of the mean values. Number of cases examined, n = 10,000.

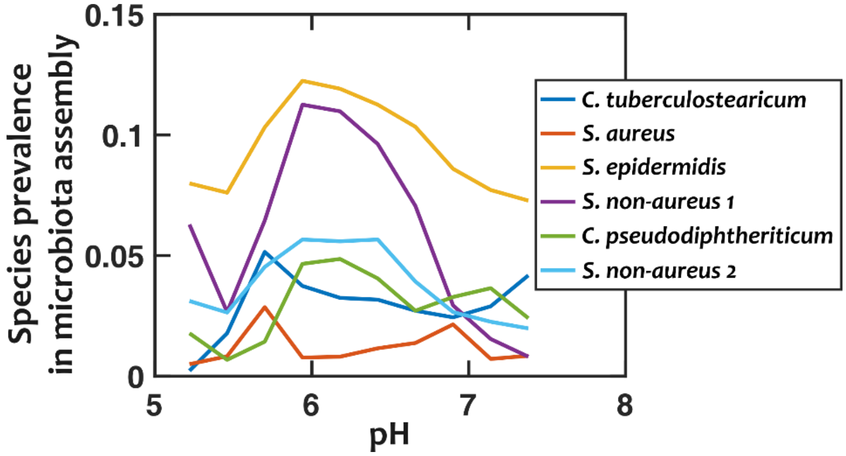

Supplementary Fig S6. Communities assembled at different pH values show distinct and pH-dependent profiles of species-level prevalence. We binned simulated communities assembled under a fixed-pH regime according the pH and within each bin assessed how often different species appeared in assembled *in silico* communities. The prevalence of species appears to depend on pH and vary from one species to another. Number of cases examined, n = 10,000.

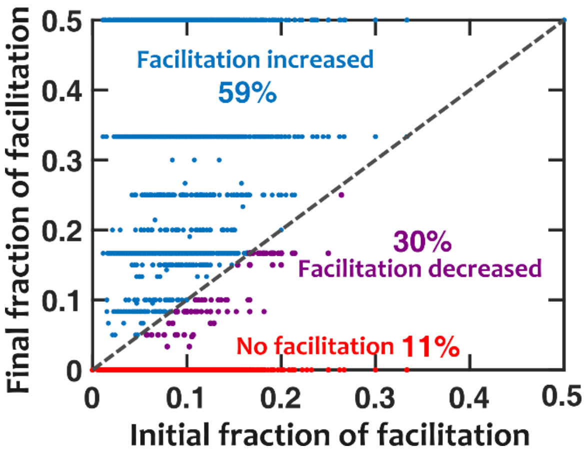

Supplementary Fig S7. Prevalence of interspecies facilitation increases in stable *in silico* communities. Using the empirically measured parameters for species growth and interactions, we assembled communities based on *in silico* strains that resembled those species. We then examined how the fraction of interactions among final assembled in silico communities was related to the pool of species before assembly. We found that among cases that contained facilitation interactions in the initial pool of species, the final stable communities were more likely (66%:34%) to contain a higher fraction of facilitation interactions. Because there is no example of mutual facilitation among our species, the final fraction of facilitation is bound to 50%. Number of cases examined, n = 10,000.

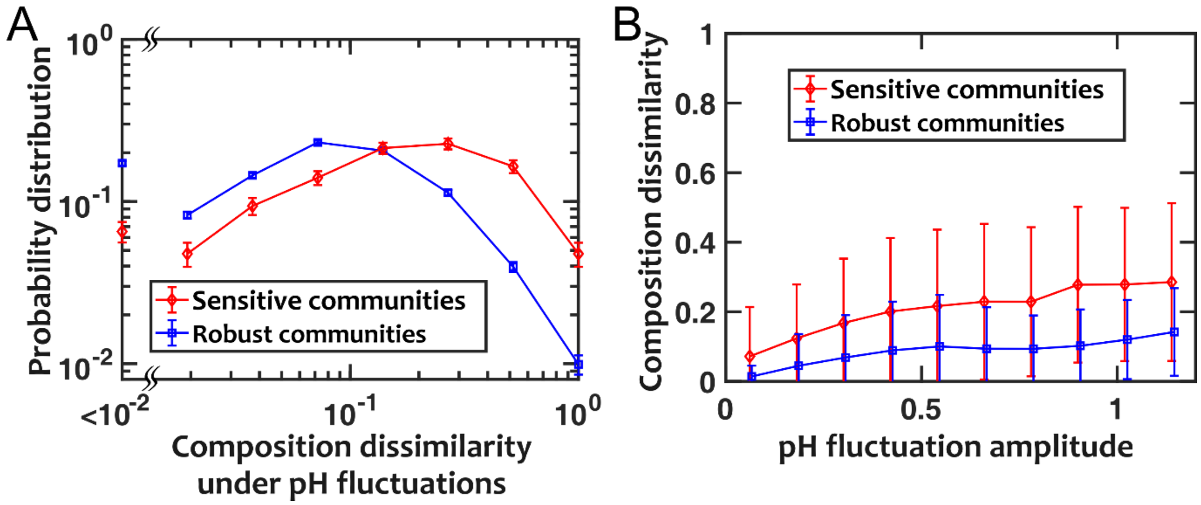

Supplementary Fig S8. *In silico* communities that are sensitive to dilution rate changes show higher deviation when exposed to fluctuation pH compared to communities that are robust to dilution rate changes. Depending on the sensitivity of community composition to changes in dilution rate, we grouped *in silico* communities to robust (<=10% dissimilarity in composition under ±50% change in dilution rate) versus sensitive (>10% dissimilarity under ±50% change in dilution rate). When these communities were exposed to a fluctuating pH, we observed that sensitive (compared to robust) communities were more prone and showed a larger dissimilarity in composition. Among both sensitive and robust communities, dissimilarity in composition increased when the amplitude of pH fluctuations increased. Number of cases examined, n = 10,000.

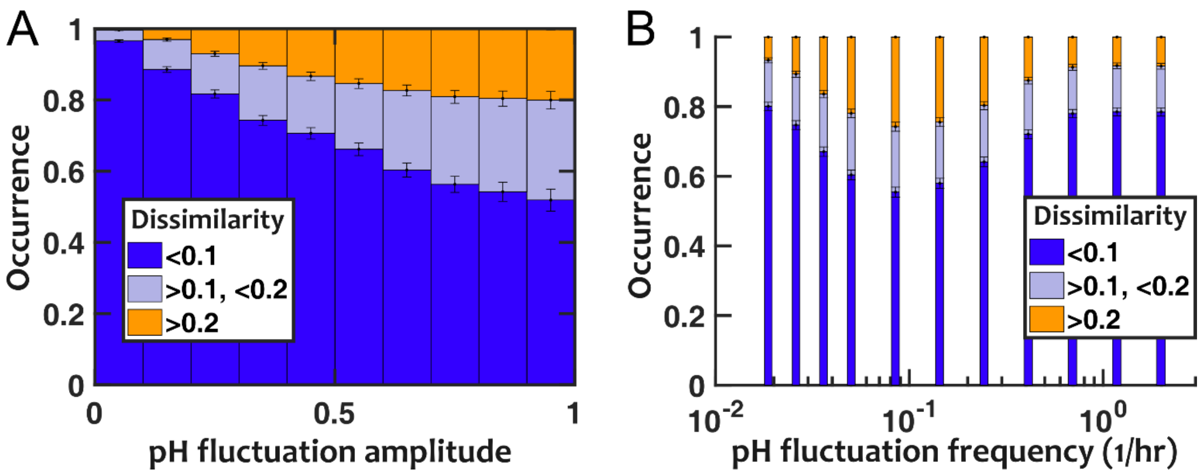

Supplementary Fig S9. A randomly fluctuating pH shows trends similar to a sinusoidal pH variation in how composition deviations (quantified using Bray-Curtis dissimilarity) depend on the frequency and amplitude of fluctuations. Unlike sinusoidal changes in pH in the main body of the paper, here pH transitions randomly between two distinct pH values, with time between transitions following an exponential probability distribution (i.e. transition times following a Poisson distribution). In these results, the average frequency of pH transitions is f_{pH} = 0.2/hr and the step between the two pH levels is ΔpH = 0.5 (on average comparable to ΔpH = 0.5 for sinusoidal fluctuations).

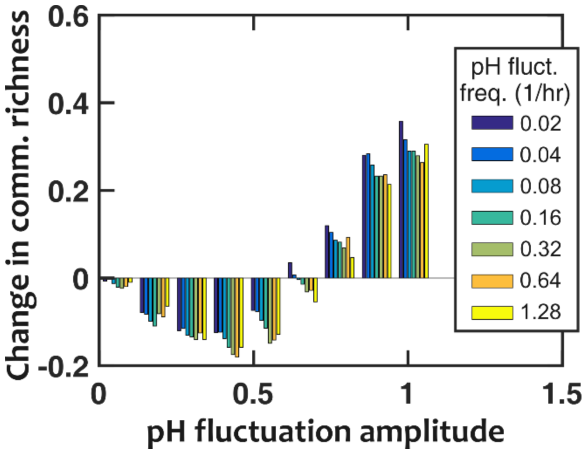

Supplementary Fig S10. If pH fluctuates during community assembly, the richness of resulting communities could be different from the fixed-pH case. However, unlike theoretical predictions, the richness does not monotonically increase when the amplitude of pH fluctuations increases. We examined fluctuations at different frequencies and observed little dependence of the outcome on the pH fluctuation frequencies. Nasal microbiota assembly is only modestly affected under pH fluctuations. Number of assembly cases at each fluctuation frequency, n = 1,000.

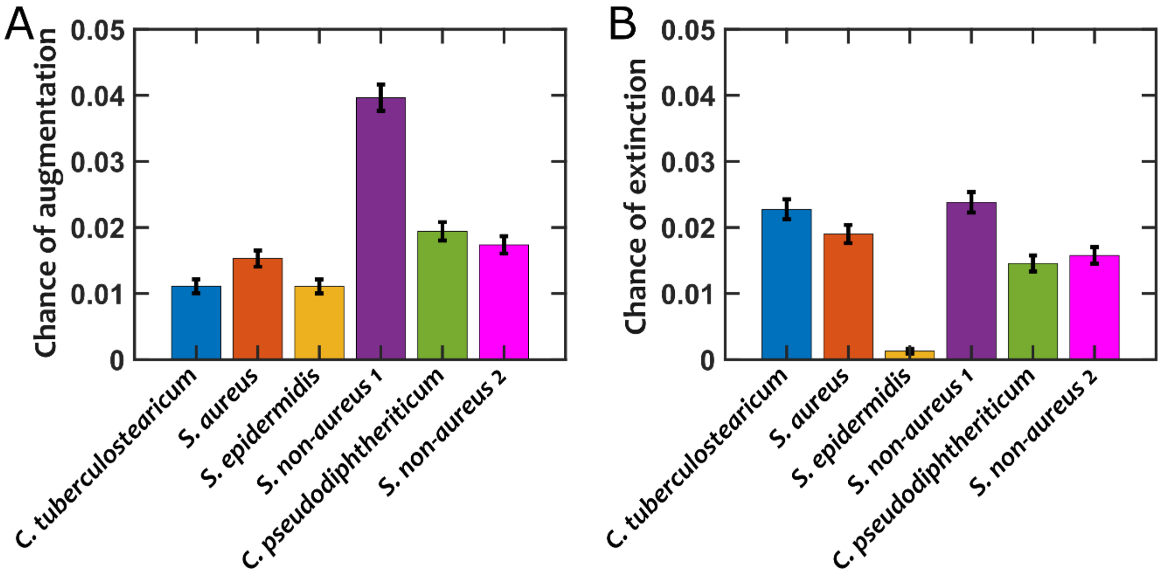

Supplementary Fig S11. Species with the most facilitation are most likely to be added into and species with the most inhibition are least likely to drop from nasal microbiota that are assembled under a fluctuating pH. We enumerated the probability at the species level that a member would be (A) added to or (B) dropped from a community during assembly when the pH fluctuated (compared to the fixed-pH case). The amplitude of pH fluctuations tested varied from 0.1 to 1 and the frequency of fluctuations tested varied from 0.02/hr to 1.28/hr (similar to Fig S10). Number of assembly cases at each fluctuation frequency, n = 1,000.

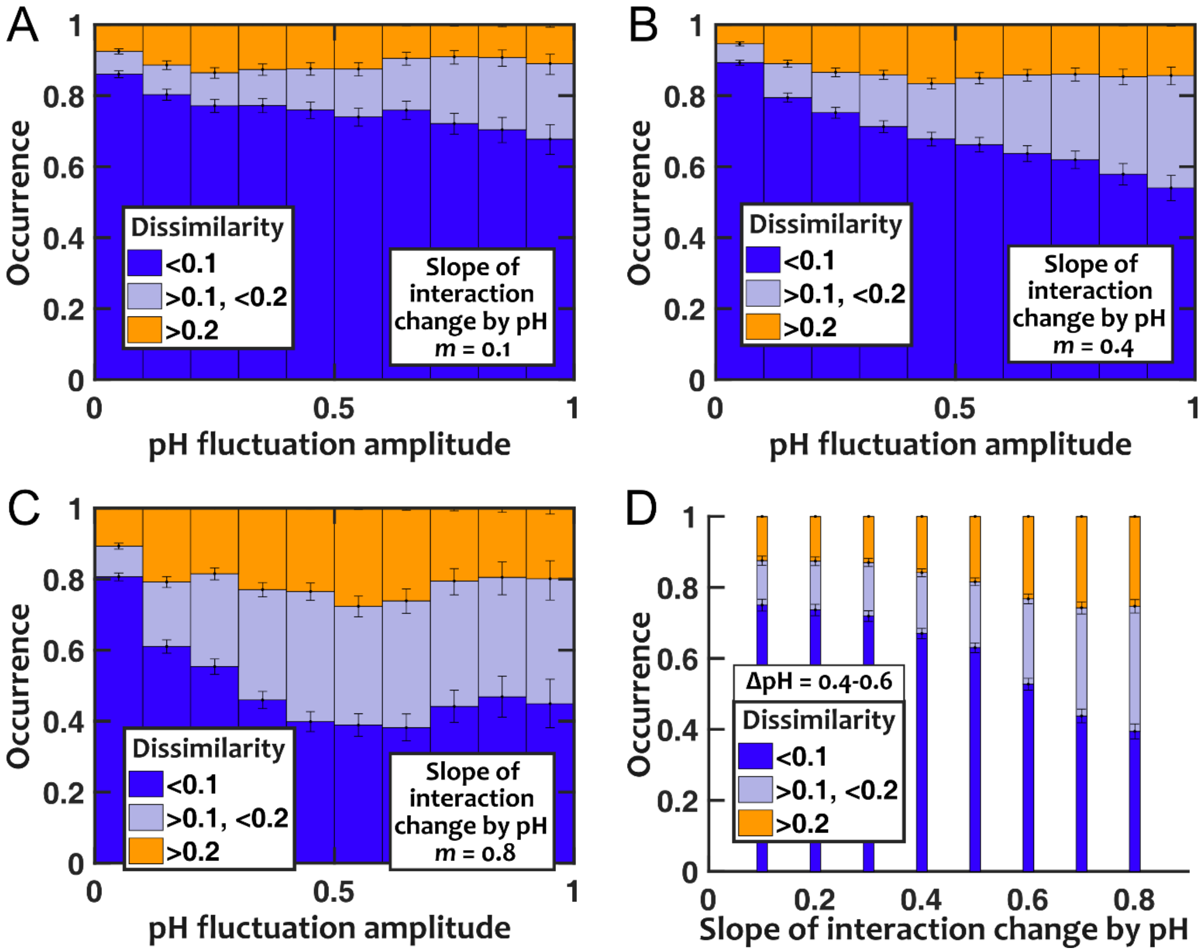

Supplementary Fig S12. With temporal fluctuations in pH, pH-dependency of interaction coefficients affect the community composition only when the dependency is very strong. We allowed interaction coefficients to each linearly change with pH with a slope randomly selected from a uniform distribution in the range [-*m*, *m*]. (A-C) Under a temporally fluctuating pH, as the strength of pH dependency of interactions (i.e. *m*) is increased, we observe more deviation from the stable community composition. For each *m* in (A-C), the composition deviates more as the amplitude of pH fluctuation increases, consistent with our results for pH-independent interactions in Fig 4. (D) Comparing the results for simulated cases with a pH fluctuation amplitude (ΔpH) between 0.4 and 0.6, we note that the effect of pH-dependent interactions on composition deviation is minimal until the dependency becomes strong enough that the change becomes comparable to the interaction coefficients themselves ($m > 0.4$). The frequency of fluctuations $f_{pH} = 0.2$/hr (similar to Fig 4). Number of communities examined, n = 10,000.
